# Depressive symptoms and associated factors among left-behind children in China: a cross-sectional study

**DOI:** 10.1186/s12889-018-5963-y

**Published:** 2018-08-23

**Authors:** Meijuan Tan, Mengshi Chen, Jing Li, Xinyun He, Zhiyong Jiang, Hongzhuan Tan, Xin Huang

**Affiliations:** 1 Department of Epidemiology and Health Statistics, School of Public Health, Central South University, Changsha, 410008 China; 2 Brains Hospital of Hunan Province, Changsha, 410007 China; 3 Department of Cardiology, the First Hospital of Changsha, Changsha, 410000 China; 4 Mental Hospital of Anhua County, Yiyang, 413500 China; 5 Department of preventive medicine, School of medicine, Hunan Normal University, Changsha, 410013 China

**Keywords:** Left-behind children, Depressive symptoms, Prevalence, Epidemiology, Junior and senior secondary schools

## Abstract

**Background:**

To investigate the prevalence of depressive symptoms among left-behind children (LBC) in junior and senior secondary schools and examine the significant predictors of depressive symptoms, which might provide practical intervention measures for the schools.

**Methods:**

By using stratified random sampling, 1076 (LBC) in junior and senior secondary schools were investigated in the study. Depressive symptoms were assessed using the depression self-rating scale (SDS). SDS raw scores 40 or higher were categorised as depressive symptoms.

**Results:**

The total prevalence of depressive symptoms was 54.74% for LBC in junior and senior secondary schools, with 73.08% for grade 12 students. The multivariate logistic regression analysis showed that grades, family income, parental relationship, parent-child relationship and teacher-student relationship were significantly associated with depressive symptoms.

**Conclusions:**

Depressive symptoms are acommon health problem among LBC in junior and senior secondary schools, and LBC in grade 12 may be at high risk of depressive symptoms. The parents, teachers and schools should pay more attention to LBC, particularly those in grade 12, and provide prevention and early intervention programs such as individual counsel service to prevent depressive symptoms.

**Electronic supplementary material:**

The online version of this article (10.1186/s12889-018-5963-y) contains supplementary material, which is available to authorized users.

## Background

Many labour populations have moved from rural to urban environments for better job opportunities in China since the 1990s. With the rapid increase in the number of labour migrants, the number of children who have been left at their original residence and separated from their parents is rising fast to about 23–30 million in rural China [1]. Such migration possibly affects the psychological status of the migrant family members [2], especially the children who are left behind. In China, the term ‘left-behind children’ (LBC) is defined as children under 18 years old who stay at their home while one or both parents migrate to other places for work without living together for at least 6 months [3]. LBC not only lack care and nutrition [4, 5], but they also have to face academic pressure and challenges from peers. More importantly, this pressure would be obvious when they enter junior and senior secondary schools. In the absence of one or both parents, LBC unavoidably have to experience to some extent a sense of helplessness and suffer from psychological problems such as depression, anxiety, and loneliness [6, 7]. Depressive symptoms can affect children’s feelings and mood directly, which is known to be a common psychological problem. In China, previous studies have indicated that the occurrence of depressive symptoms in LBC range from 12.10% in a county of the Anhui province [8] to 63.75% in Yanji City in Jilin [9]. Depressive symptoms were significantly associated with age, gender, grade, parental education and occupation, place of residence, income situation, parental relationship, lifestyle habits, physical activity and sleep dissatisfaction [10–12]. To some extent, the wide range of the estimated prevalence of depressive symptoms among LBC may be the result of different sociocultural characteristics of particular study populations, the methodological approaches and the psychometric properties of the tools used. Knowing the prevalence and associated factors of depressive symptoms can encourage education departments to pay more attention to the mental health status among LBC and provide mental health services to them.

Previous studies of depressive symptoms of LBC in China mainly focus on the comparison between the LBC and not-left-behind children (NLBC). Moreover, the populations in previous studies were LBC in primary schools and/or junior secondary schools. In this study, we investigated the prevalence of depressive symptoms among LBC in junior and senior secondary schools and examined the significant predictors of depressive symptoms, which might provide practical intervention measures for the schools.

## Methods

### Study population

A cross-sectional, descriptive study was conducted between October 2011 and April 2012 in Anhua County, Hunan Province. Anhua County covers a geographic area of 4950 km^2^, includes 23 towns and has a population of around 1.03 million people, with 34,020 adolescent children in junior and senior secondary schools in 2011. There were 24 junior secondary schools for children aged 11–16 years and 10 senior secondary schools for those aged 14–18 years. All LBC in the selected junior (grades 7–9) and senior (grades 10–12) secondary schools were invited to join the study.

A stratified, two-stage cluster sampling was used. Twenty-four junior secondary schools and 10 senior secondary schools in Anhua County were the primary sampling units. In the first stage, we selected nine junior secondary schools and four senior secondary schools by random number. In the second stage, 30 classes in junior secondary schools and 68 classes in senior secondary schools were selected randomly. LBC is defined as children under 18 years old who stay at their home while one or both parents migrate to other places for work without living together for at least 6 months [3].Children who came from single-parent families and orphans were excluded. All LBC (1086) studying in the selected classes were eligible to participate in the study. Finally, we investigated 1076 LBC (with a response rate of 99.08%) with completed data for this analysis.

In our pilot testing result, the prevalence of depressive symptoms amongst LBC was 44.40%. Assuming the prevalence of depressive symptoms is 44.00%, d = 0.1 × p, *α*= 0.05, the simple sample size was calculated to be 509 subjects. Considering the design effect, the cluster sample size is about 1.5 times of the simple sample size. Therefore, the minimum sample size was calculated to be 764 subjects. In the pilot, the same questionnaire was used with the formal investigation, and the validity of the questionnaire was pretested.

### Measure

Demographic information included students’ gender, age, class, grade, school, their substance abuse and whether their parents had migrated to another place over the past 6 months. The questionnaire contained family income, social relationships (from parents, teachers and peers), academic stressors and other interest areas. All the above-mentioned variables were explained by trained data collectors when conducting the investigation.

Family income is defined as the income of all members of the family. High family income means the income is equal to or more than 5000 RMB a month (≥5000); middle family income means the income is equal to or more than 3000 and less than 5000 a month (3000 ≤ family income< 5000); poor family income means the income is less than 3000 a month (< 3000).

Parental relationship is defined as the relationship between father and mother. Good parental relationship means the father and mother care and support each other, and the frequencies of their communication are equal to or more than 4 times a month. Middle parental relationship means the frequencies are 2–3 times a month. Poor parental relationship means the frequency is equal to or less than one time a month.

Parent-child relationship is defined as the relationship between children and parents. Good parent-child relationship means the parents care, support and are close to their children, and the frequencies of telephonic interactions between them are equal to or more than 4 times a month. Middle parent-child relationship means the frequencies are 2–3 times a month. Poor parent-child relationship means the frequency is equal to or less than one time a month.

Teacher-student relationship is defined as the relationship between teachers and students. Good teacher-student relationship means the teachers care and support the student, and the frequencies of their communication are equal to or more than 2 times a month. Middle teacher-student relationship means the frequency is one time a month. Poor teacher-student relationship means the frequency is less than one time a month.

Peer relationship is defined as the relationship between peers. Good peer relationship means their peers trust and support them, and the frequencies of their communication are equal to or more than 2 times weekly. Middle peer relationship means the frequency is one time weekly. Poor peer relationship means the frequency of communication is less than one time weekly.

Depressive symptoms were assessed by the Zung Self-Rating Depression Scale (SDS) (Zung WW 1965). The SDS is a self-report measure of depression, which consists of 20 items that cover affective, psychological and somatic features of depression. Each item is ranked from 1 to 4, and a higher score indicates a greater degree of depressive symptoms, making an overall score range from 20 to 80. All subjects were categorised into two groups: (1) Non-depressive symptoms group (SDS < 40) and (2) Depressive symptoms group (SDS ≥ 40). The cut of values is established based on the SDS (Zung WW 1965). The SDS has been developed and proven to be quantitatively adequate for the subjective perception of depression and has good validity and reliability in China; the Cronbach’s alpha coefficient and test-retest reliability are 0.862 and 0.820, respectively.

### Data analysis

All analyses were performed with the Statistical Package for Social Sciences software (SPSS-version 18). The statistical tests were two-tailed, with *p* values ≤0.05 being considered statistically significant. Descriptive statistics were calculated for sociodemographic characteristics and depressive symptoms of LBC and were presented appropriately in frequencies, mean values and standard deviation. Between-group comparisons for discontinuous variables were done by the Chi-square test. In logistic regression analysis, the dependent variable was depression symptoms group with the SDS raw score 40 or higher, and the covariates included gender, grades, family income, parental relationship, parent-child relationship, teacher-student relationship and peer relationship which were statistically significantly associated with depression in the univariate analyses. The group with lowest prevalence of depression symptoms in the univariate analysis was considered as the reference group. Logistic regression (backward method: LR *α*in = 0.05, *α*out = 0.10) was conducted to assess the relationship between possible associated variables and the symptoms of depression, expressed as odds ratios (OR) with 95% confidence interval (CI).

## Results

### Sociodemographic characteristics of the participants

**Fig. 1:**
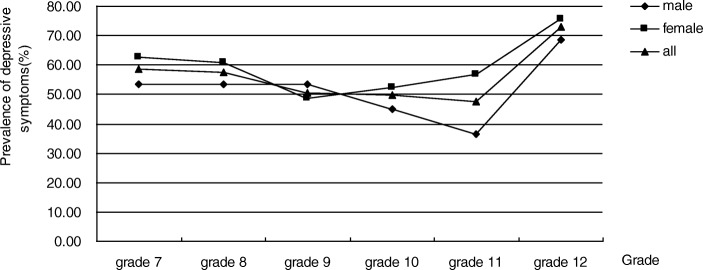
Prevalence of depressive symptoms across grade of LBC

The final sample consisted of 1076 participants from the selected junior and senior secondary schools, of whom 631 (58.64%) were female and 445 (41.36%) were male. The mean age of the participants was 14.34 (SD =1.72, range: 11–18). See Table 1. The mean (±SD) SDS raw score was 40.57 ± 7.51, ranging from 20 to 68. Out of all participants, 589 LBC SDS raw scores were 40 or higher, yielding the prevalence rate of depressive symptom at 54.74%. It was 62.10% from participants with low family income. The prevalence of depressive symptoms was 73.08% in grade 12, whereas it was 47.73% in grade 11 (Additional file 1: Table S1). In grade 12, the prevalence of depressive symptoms was 68.42% and 75.76% among male and female participants, respectively (Fig. 1).

**Table 1:** The description of left-behind children on basic characteristics (*n* = 1076)

Variables	Frequency (N)	Percentage (%)
Age		
11–15	606	56.32
15–18	470	43.68
Gender		
Male	445	41.36
Female	631	58.64
Grades		
Grades 7	291	27.04
Grades 8	218	20.26
Grades 9	220	20.45
Grades 10	163	15.15
Grades 11	132	12.27
Grades 12	52	4.83
Family income		
High	33	3.07
Middle	795	73.88
Low	248	23.05
Parental relationship		
Good	670	62.27
Middle	321	29.83
Poor	85	7.90
Parents’ expectation		
High	626	58.18
Middle	441	40.99
Low	9	0.84
Academic stressor		
Low	16	1.49
Middle	528	49.07
High	532	49.44
Parent-child relationship		
Good	444	41.26
Middle	590	54.83
Poor	42	3.90
Teacher-student relationship		
Good	545	50.65
Middle	504	46.84
Poor	27	2.51
Peer relationship		
Good	721	67.01
Middle	344	31.97
Poor	11	1.02
Substance abuse		
No	955	88.75
Yes	121	11.25

### The association between associated variables and SDS

Table 2 show that the prevalence of depressive symptoms in LBC was significantly associated with parental relationship, parent-child relationship, and teacher-student relationship. LBC with middle/poor parental relationship, parent-child relationship, teacher-student relationship and middle peer relationship had significantly higher prevalence of depressive symptoms than the reference group. See Table 2.

**Table 2:** Univariate logistic regression analysis of the factors associated with depressive symptoms in left-behind children (*n* = 1076)

Variables	N	Depressed	rate (%)	OR	OR (95%CI)
Parental relationship					
Good	670	325	48.51	1(reference)	
Middle	321	204	63.55	1.85	1.41–2.43
Poor	85	60	70.59	2.55	1.56–4.16
Parents’ expectation					
High	626	329	52.56	1(reference)	
Middle	441	253	57.37	1.22	0.95–1.55
Low	9	7	77.78	3.16	0.65–15.33
Academic stressor					
Low	16	8	50.00	1(reference)	
Middle	528	270	51.14	1.05	0.39–2.83
High	532	311	58.46	1.41	0.52–3.81
Parent-child relationship					
Good	444	202	45.50	1(reference)	
Middle	590	360	61.02	1.88	1.46–2.41
Poor	42	27	64.29	2.16	1.12–4.17
Teacher-student relationship					
Good	545	244	44.77	1(reference)	
Middle	504	323	64.09	2.20	1.72–2.82
Poor	27	22	81.48	5.43	2.03–14.54
Peer relationship					
Good	721	359	49.79	1(reference)	
Middle	344	221	64.24	1.81	1.39–2.36
Poor	11	9	81.82	4.54	0.97–21.15
Substance abuse					
No	955	514	53.82	1(reference)	
Yes	121	75	61.98	1.40	0.95–2.06

### The results of multivariate logistic regression analysis

The results of the multivariate logistic regression analysis (Table 3) showed that grades, family income, parental relationship, parent-child relationship and teacher-student relationship were significantly associated with depressive symptoms. Children in grade 12 had a 3.25 higher odds of depressive symptoms compared to their peer in grade 11. Children from low family income had a 2.66 higher odds of depressive symptoms compared to their peer from high family income. Poor parental relationship, middle parent-child relationship and poor teacher-student relationship increased the odds of depressive symptoms among LBC (OR = 1.87, *P* < 0.05, OR = 1.52, *P* < 0.01, and OR = 4.16, *P* < 0.01, respectively).

**Table 3:** The results of multivariate logistic regression analysis

Variables	B	Wald	*p*	OR	95%CI
Gender					
Male				1(reference)	
Female	0.243	3.345	0.067	1.28	0.98–1.65
Grades		19.550	0.002		
Grades 11				1(reference)	
Grades 7	0.669	8.828	0.003	1.95	1.26–3.04
Grades 8	0.452	3.656	0.056	1.57	0.99–2.50
Grades 9	0.301	1.674	0.196	1.35	0.86–2.13
Grades 10	0.064	0.068	0.794	1.07	0.66–1.73
Grades 12	1.179	10.009	0.002	3.25	1.57–6.75
Family income		7.316	0.026		
High				1(reference)	
Middle	0.672	3.056	0.080	1.96	0.92–4.16
Low	0.977	5.866	0.015	2.66	1.21–5.85
Parental relationship		10.189	0.006		
Good				1(reference)	
Middle	0.400	7.035	0.008	1.49	1.11–2.00
Poor	0.625	5.349	0.021	1.87	1.10–3.17
Parent-child relationship		9.034	0.011		
Good				1(reference)	
Middle	0.420	9.017	0.003	1.52	1.16–2.00
Poor	0.329	0.828	0.363	1.39	0.68–2.82
Teacher-student relationship		22.473	0.000		
Good				1(reference)	
Middle	0.585	17.621	0.000	1.79	1.37–2.36
Poor	1.426	7.463	0.006	4.16	1.50–11.58
Peer relationship		5.581	0.061		
Good				1(reference)	
Middle	0.307	4.348	0.037	1.36	1.02–1.81
Poor	1.006	1.519	0.218	2.73	0.55–13.53

## Discussion

Previous studies on depressive symptoms of school-aged LBC in China focus on the primary schools or/and junior secondary schools. Few studies have reported the prevalence and its associated factors of depressive symptoms among LBC in junior and senior secondary schools. Depressive symptoms are a common health problem among LBC in the study, and LBC in grade 12 may be at high risk of depressive symptoms. Grades, family income, parental relationship, parent-child relationship, teacher-student relationship, and peer relationships were significantly associated with depressive symptoms.

Our study showed that 54.74% of LBC had depressive symptoms among junior and senior secondary school students, which was different with the results in previous studies among different populations [9, 13]. The prevalence of depressive symptoms among LBC was 24.79% in Chongqing, a western city of China [14]; 40.37% in Guangxi, a southern province of China [10]; and 63.75% in Yanji City of Jilin, a northern city of China [9]. To some extent, the wide range of estimates of the prevalence of depressive symptoms might be explained by the variety of the methodological approaches, psychometric tools or particular study populations. A fascinating finding from our study is that the prevalence of depressive symptoms among grade 12 students was significantly higher than the overall and in other grades. Students in grade 12 had a 3.25 higher odds of depressive symptoms than their peers in grade 11. The result showed the LBC of grade 12 were more vulnerable to depressive symptoms. This is more likely to be because LBC in grade 12 need to participate in the college entrance examination. In China, teachers, parents and students pay more attention to the college entrance examination and consider it a golden opportunity to change their lives. Moreover, LBC have more stress than NLBC because their parents have higher expectations of them and make more effort to better their future. Thus, their mental health might be more negatively influenced by the prospect of the examination.

In the multivariate logistic regression analysis, our study showed that the relationship between gender and depressive symptoms was not significant, although female LBC manifested higher levels than males. However, some studies reported statistically significant differences in depressive symptoms by gender [11, 15, 16]. The relationship between gender and depressive symptoms need to be explored in further studies. The significant associations of family income with depressive symptoms are consistent with previous studies [17]. The present study revealed that LBC from poor families had higher levels of depressive symptoms than those from well-off families, with a 2.66 higher odds than their peers from high family income. Low income may affect people’s self-esteem, self-confidence and their sensitivities about social status. Generally, those regarding themselves as having low-income status were pessimistic about life and exhibited high levels of depressive symptoms. However, previous studies indicate either no differences or the opposite pattern, with the students living with rich parents more likely to be depressed [11, 18]. The relationship between family income and depressive symptoms needs to be explored in further studies.

In this study, parental relationship was related to the development of depressive symptoms among LBC. Based on previous literature, problematic parental relationships constitute additive risk factors that directly and independently foster depressive symptoms, especially inter-parental conflicts [19]. It may be that parents spend a great deal of time to address the problematic relationship between themselves, thus, pay relatively little attention to their children’s lives and emotional or behavioural problems. In addition, a good parental relationship improves the level of care for children to prevent depressive symptoms among LBC [12].

The present study indicated a significant association between the parent–child relationships and the prevalence of depressive symptoms among LBC. Parents bestow their provisions as well as care, security, and support on their children, which are effective protectors and buffers for children against depressive symptoms [20]. Numerous studies report that a good quality of parent-child relationship promotes positive adjustment and decreases the vulnerability to depressive symptoms among different populations [21, 22]. To a great extent, parental absence not only affects adversely the love and attention the children need, but also the level of closeness within the parent-child relationship. Similar findings have also been reported among university students [23]. Similar association was observed between teacher-student relationships and the prevalence of depressive symptoms. The teacher-student relationship is believed to be the expansion of the parent-child relationship and play an important role in students’ experience, particularly when they are separated from their parents. A positive teacher-student relationship can protect adolescents against depressive symptoms [24]. Consistent with numerous researches in other populations [23, 25], our study indicated significant associations between peer relationships and the prevalence of depressive symptoms among LBC. Positive peer relationships that are characterized by trust and supportiveness have been thought to enhance positive perceptions of the school climate for students. Moreover, prior research indicates that students who lack support from close peers have been predicted to develop increased symptoms of depression [18]. LBC have to spend more time with their peers than with their parents, and they need trust and supportiveness from their peers.

There is a vast amount of evidence across countries of the association of substance abuse with depressive symptoms [11, 26], but we did not observe this evidence in our study. This difference may result from traditional education and self-control.

The strengths of this study are large random sample size with a satisfactory response rate. However, this study is a cross-sectional study that may only suggest but not determine causality. Prospective cohort and longitudinal studies are needed to assess depressive symptoms among LBC. Like other self-rating scales, the self-reported scale may be reporting bias. Nonetheless, participation was voluntary and anonymous to guarantee the validity of information. The Zung Self-rating depression scale was originally developed for patients, but it had been widely used to assess depressive symptoms for apparently healthy school children and adolescents. Additionally, we evaluated the reliability and validity of the Zung Self-rating scale on depression for apparently healthy school children. Few variables such as lifestyle habits, physical activity, sleep satisfaction psychiatric history of family, and parental education were not included in the study.

## Conclusions

Depressive symptoms are a common health problem among left-behind adolescent children in junior and senior secondary schools, and LBC in grade 12 may be at particularly high risk of developing depressive symptoms. LBC who are female, with low family income, poor parental relationship, middle parent-child relationship, poor teacher-student relationship and middle peer relationship are considered at higher risk for depressive symptoms. For LBC, their parents should remain close to them and take care of their mental health, especially depressive symptoms. The teachers should provide more care and support to LBC and popularize the knowledge about depressive symptoms to LBC, particularly those in grade 12. Parents and teachers should communicate with LBC as much as possible. The schools should pay more attention to LBC and provide educational programs in positive health strategies and school counselling programs, such as education workshops, symposia, individual counselling, to reduce and even prevent depressive symptoms. Moreover, targeted interventions to LBC would empower them to develop coping strategies against depressive symptoms.

## Additional file

Additional file 1: The association between sociodemographic characteristics and SDS in left-behind children (*n* = 1076). The prevalence of depressive symptoms in LBC was significantly associated with gender, grades and family income. (DOC 113 kb)

## Abbreviations

CI: Confidence interval
LBC: Left-behind children
NLBC: Not-left-behind children
OR: Odds ratios
SD: Standard Deviation
SDS: The Depression Self-rating Scale

## References

[1] Duan CR, Zhou FL (2005). Research on the left-behind children in China (in Chinese). Popul Res.

[2] Lu Y, Hu P, Treiman DJ (2012). Migration and depressive symptoms in migrant-sending areas: findings from the survey of internal migration and health in China. Int J Public Health.

[3] Zhou FL, Duan CR (2006). A review on Left-behind Children (in Chinese). Popul J.

[4] Luo J, Peng X, Zong R, Yao K, Hu R (2008). The status of care and nutrition of 774 left-behind children in rural areas in China. Public Health Rep.

[5] Tan C, Luo J, Zong R, Fu C, Zhang L (2010). Nutrition knowledge, attitudes, behaviours and the influencing factors among non-parent caregivers of rural left-behind children under 7 years old in China. Public Health Nutr.

[6] Graham E, Jordan LP (2011). Migrant parents and the psychological well-being of left-behind children in Southeast Asia. J Marriage Fam.

[7] Wickramage K, Siriwardhana C, Vidanapathirana P, Weerawarna S, Jayasekara B (2015). Risk of mental health and nutritional problems for left-behind children of international labor migrants. BMC Psychiatry.

[8] Wang Tiezhu CM, Yehuan S (2011). Research on children's depression and the influence of left-behind status in rural area (in Chinese). Chin J Sch Health.

[9] Chenggao Z, Xiuying Y (2009). Study on depressive symptoms and its relative factors of the Korean-Chinese students in junior high school (in Chinese). J Med Sci Yanbian Univ.

[10] Lan YL, Yan L, Tang XJ (2009). Personality and depressive symptoms and their influential factors in children left-behind in rural area (in Chinese). Chin J Public Health.

[11] Park HY, Heo J, Subramanian SV, Kawachi I, Oh J (2012). Socioeconomic inequalities in adolescent depression in South Korea: a multilevel analysis. PLoS One.

[12] Chen L, Wang L, Qiu XH, Yang XX, Qiao ZX (2013). Depression among Chinese university students: prevalence and socio-demographic correlates. PLoS One.

[13] He J, Ma H, Xu L-z (2011). Investigation on depression status among left-behind children in rural areas of Anhui Province (in Chinese). Prev M ed T rib.

[14] Wang L, Feng Z, Yang G, Yang Y, Dai Q (2015). The epidemiological characteristics of depressive symptoms in the left-behind children and adolescents of Chongqing in China. J Affect Disord.

[15] Al-Faris EA, Irfan F, Van der Vleuten CP, Naeem N, Alsalem A (2012). The prevalence and correlates of depressive symptoms from an Arabian setting: a wake up call. Med Teach.

[16] Sokratous S, Merkouris A, Middleton N, Karanikola M (2013). The association between stressful life events and depressive symptoms among Cypriot university students: a cross-sectional descriptive correlational study. BMC Public Health.

[17] Herrmann J, Vogel M, Pietzner D, Kroll E, Wagner O (2018). Factors associated with the emotional health of children: high family income as a protective factor. Eur Child Adolesc Psychiatry.

[18] Chango JM, McElhaney KB, Allen JP, Schad MM, Marston E (2012). Relational stressors and depressive symptoms in late adolescence: rejection sensitivity as a vulnerability. J Abnorm Child Psychol.

[19] Cummings EM, Koss KJ, Davies PT (2015). Prospective relations between family conflict and adolescent maladjustment: security in the family system as a mediating process. J Abnorm Child Psychol.

[20] Sund AM, Wichstrom L (2002). Insecure attachment as a risk factor for future depressive symptoms in early adolescence. J Am Acad Child Adolesc Psychiatry.

[21] Hazel NA, Oppenheimer CW, Technow JR, Young JF, Hankin BL (2014). Parent relationship quality buffers against the effect of peer stressors on depressive symptoms from middle childhood to adolescence. Dev Psychol.

[22] Babore A, Trumello C, Candelori C, Paciello M, Cerniglia L (2016). Depressive symptoms, self-esteem and perceived parent-child relationship in early adolescence. Front Psychol.

[23] Lee RB, Sta Maria M, Estanislao S, Rodriguez C (2013). Factors associated with depressive symptoms among Filipino university students. PLoS One.

[24] Wang MT, Brinkworth M, Eccles J (2013). Moderating effects of teacher-student relationship in adolescent trajectories of emotional and behavioral adjustment. Dev Psychol.

[25] Narr RK, Allen JP, Tan JS, Loeb EL. Close friendship strength and broader peer group desirability as differential predictors of adult mental health. Child Dev. 2017; 10.1111/cdev.12905. 10.1111/cdev.12905 PMC5821600 28832975

[26] Igwe WC, Ojinnaka NC (2010). Mental health of adolescents who abuse psychoactive substances in Enugu, Nigeria - a cross-sectional study. Ital J Pediatr.

