# Supplementary material for: Depressive symptoms and associated factors among left-behind children in China: a cross-sectional study
Source: BMC Public Health. 2018 Aug 23;18:1059. doi: 10.1186/s12889-018-5963-y (PMC6108138; doi:10.1186/s12889-018-5963-y)
Supplement: Supplementary file 1 — The association between sociodemographic characteristics and SDS in left-behind children (n = 1076). The prevalence of depressive symptoms in LBC was significantly associated with gender, grades and family income. (DOC 113 kb) [file 12889_2018_5963_MOESM1_ESM.doc]

The association between sociodemographic characteristics and SDS in left-behind children (n=1076)

| Variables | N | Depressed | rate（%） | chi-square test | p-value |
| --- | --- | --- | --- | --- | --- |
| Age |  |  |  |  |  |
| 11-15 | 606 | 337 | 55.61 | 0.425 | 0.515 |
| 15-18 | 470 | 252 | 53.62 |  |  |
| Gender |  |  |  |  |  |
| Male | 445 | 226 | 50.79 | 4.787 | 0.029 |
| Female | 631 | 363 | 57.53 |  |  |
| Grades |  |  |  |  |  |
| Grades 7 | 291 | 171 | 58.76 | 15.479 | 0.008 |
| Grades 8 | 218 | 125 | 57.34 |  |  |
| Grades 9 | 220 | 111 | 50.45 |  |  |
| Grades 10 | 163 | 81 | 49.69 |  |  |
| Grades 11 | 132 | 63 | 47.73 |  |  |
| Grades 12 | 52 | 38 | 73.08 |  |  |
| Family income |  |  |  | 10.669 | 0.005 |
| High | 33 | 12 | 36.36 |  |  |
| Middle | 795 | 423 | 53.21 |  |  |
| Low | 248 | 154 | 62.10 |  |  |
| Total | 1076 | 589 | 54.74 |  |  |
